# Auditory Cortex Tracks Both Auditory and Visual Stimulus Dynamics Using Low-Frequency Neuronal Phase Modulation

**DOI:** 10.1371/journal.pbio.1000445

**Published:** 2010-08-10

**Authors:** Huan Luo, Zuxiang Liu, David Poeppel

**Affiliations:** 1 State Key Laboratory of Brain and Cognitive Science, Institute of Biophysics, Chinese Academy of Sciences, Beijing, China; 2 Department of Psychology, New York University, New York, New York, United States of America; McGill University, Canada

## Abstract

Integrating information across sensory domains to construct a unified representation of multi-sensory signals is a fundamental characteristic of perception in ecological contexts. One provocative hypothesis deriving from neurophysiology suggests that there exists early and direct cross-modal phase modulation. We provide evidence, based on magnetoencephalography (MEG) recordings from participants viewing audiovisual movies, that low-frequency neuronal information lies at the basis of the synergistic coordination of information across auditory and visual streams. In particular, the phase of the 2–7 Hz delta and theta band responses carries robust (in single trials) and usable information (for parsing the temporal structure) about stimulus dynamics in both sensory modalities concurrently. These experiments are the first to show in humans that a particular cortical mechanism, delta-theta phase modulation across early sensory areas, plays an important “*active*” role in continuously tracking naturalistic audio-visual streams, carrying dynamic multi-sensory information, and reflecting cross-sensory interaction in real time.

## Abstract 2

How is naturalistic multisensory information combined in the human brain? Based on MEG data we show that phase modulation of visual and auditory signals captures the dynamics of complex scenes.

## Author Summary

When faced with ecologically relevant stimuli in natural scenes, our brains need to coordinate information from multiple sensory systems in order to create accurate internal representations of the outside world. Unfortunately, we currently have little information about the neuronal mechanisms for this cross-modal processing during online sensory perception under natural conditions. Neurophysiological and human imaging studies are increasingly exploring the response properties elicited by natural scenes. In this study, we recorded magnetoencephalography (MEG) data from participants viewing audiovisual movie clips. We developed a phase coherence analysis technique that captures—in single trials of watching a movie—how the phase of cortical responses is tightly coupled to key aspects of stimulus dynamics. Remarkably, auditory cortex not only tracks auditory stimulus dynamics but also reflects dynamic aspects of the visual signal. Similarly, visual cortex mainly follows the visual properties of a stimulus, but also shows sensitivity to the auditory aspects of a scene. The critical finding is that cross-modal phase modulation appears to lie at the basis of this integrative processing. Continuous cross-modal phase modulation may permit the internal construction of behaviorally relevant stimuli. Our work therefore contributes to the understanding of how multi-sensory information is analyzed and represented in the human brain.

## Introduction

We do not experience the world as parallel sensory streams; rather, the information extracted from different modalities fuses to form a seamlessly unified multi-sensory percept dynamically evolving over time. There is a compelling benefit to multimodal information: behavioral studies show that combining information across sensory domains enhances unimodal detection ability—and can even induce new, integrated percepts [1]–[4]. The relevant neuronal mechanisms have been widely investigated. One typical view posits that multisensory integration occurs at later stages of cortical processing, subsequent to unisensory analysis. This view has been supported by studies showing that higher, “association” areas in temporal, parietal, and frontal cortices receive inputs from multiple unimodal areas [5]–[8] and respond to stimulation in manner that reflects multisensory convergence, for example with amplified or suppressed responses for multimodal over unimodal stimuli [9]–[12].

A growing body of evidence provides a complementary view, suggesting that cross-modal interaction is not restricted to association areas and can occur at early, putatively unisensory cortical processing stages [11],[13]. For example, non-auditory stimulation (visual and somatosensory) has been found to drive auditory cortical activity, as observed in both humans and animals [4],[14]–[23]. Similarly, visual cortical responses are modulated by inputs from other modalities [24],[25]. Importantly, independent anatomical evidence also reveals direct connections among early sensory areas [26],[27]. Therefore, multisensory integration may operate through lateral cross-sensory modulation, and there exist multiple integration pathways beyond purely hierarchical convergence [12],[28],[29].

How is early cortical activity coordinated? Beyond the classical examination of cross-modal influences on neuronal firing rate, recent studies suggest temporal coherence [30],[31] to underlie multisensory integration [28],[32]. This view posits that oscillations synchronous across different brain areas might serve an essential role in multisensory binding, similarly as that for feature binding and attentional selection [30],[33]–[36]. Several EEG/MEG studies in humans implicate oscillations and cross-area coherence in multisensory integration [29],[37]–[42]. However, most of the studies employed short, transient multisensory stimuli and focused on the evoked transient oscillatory power instead of examining sustained cross-modal modulation for long, naturalistic audiovisual streams.

Importantly, with regard to the cross-area modulation mechanism, it has recently been suggested that *cross-sensory phase modulation* may underlie this interaction [28],[32],[43],[44]. For example, non-auditory inputs (re)set the phase of ongoing local neuronal activity in auditory cortex to a high-excitability state (reflected in phase angle), effectively “selecting” or amplifying the response to subsequent auditory inputs [11],[13],[20],[22],[45]. Whether such a mechanism is implemented in populations of neurons and could mediate the perception of audiovisual speech in human viewers/listeners is completely unknown.

In order to test directly the proposal of cross-modal phase modulation of oscillatory neural activity, we investigate online audiovisual interaction, in auditory and visual cortices simultaneously, by recording magnetoencephalography (MEG) responses from human participants presented with 30-s-long natural movie clips from the movie “Dumb and Dumber” (1994, New Line Platinum Series). These video segments had either “matched” (congruent audio-visual combinations, V1A1, V2A2, V3A3) or “mixed” streams (incongruent audio-visual, V1A3, V2A1, V3A2). Building on our previous results showing that the theta-band phase pattern in human *auditory* cortex reflects the dynamic structure of spoken sentences [46], we employed a new trial-by-trial phase tracking analysis to explore multi-sensory integration. We conjectured that, in response to naturalistic audio-visual streams (movies), the low-frequency phase of auditory and visual sensory activity *in single trials* (i) will robustly track and discriminate (in a classification analysis) the sensory stream dynamics in each modality (“within-modality tracking”; i.e. auditory channel tracks auditory, visual tracks visual dynamics), (ii) may carry information about stimulus dynamics in the other modality (“cross-modality tracking”; e.g. an auditory channel can reflect visual dynamics), and (iii) that the efficacy of such cross-sensory phase modulation (trial-to-trial phase variance) depends on the relative audiovisual timing, such that a temporally matched audio-visual stream will enhance phase tracking reliability, compared to unmatched (mixed) pairs. Our data support these predictions, highlighting the critical role of cross-sensory phase modulation of oscillations in multisensory integration, commensurate with the hypothesis [28],[44]. We thus argue that multi-sensory integration may use cross-modal phase modulation as a basic mechanism to construct temporally aligned representations that facilitate perceptual decoding of audiovisual speech.

## Results

### Low-Frequency Phase Patterns in Auditory *and* Visual Areas Carry Reliable Information about Audiovisual Movies

**Figure 1:**
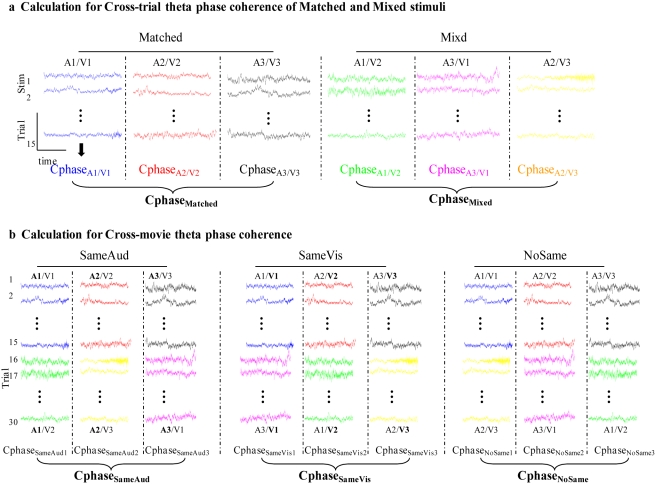
Schematic illustrating experimental design and phase coherence analysis (for a single MEG channel). The colors represent single-trial responses to each of the six audiovisual streams. The coherence analyses are performed on each of the 157 MEG channels separately. (a) The cross-trial phase coherence is calculated on all 15 trials of the same stimulus condition (same color) and compared to a mixture of trials (see Methods) to get the phase-based and power-based movie discrimination ability (see Figure 2ab). (b) Cross-movie phase coherence is calculated by combining response trials across two movie stimuli (two different colors in each column), where one dimension is matched in auditory (SameAud), visual (SameVis), or neither modality input (NoSame). See more equation details in Methods.

**Figure 2:**
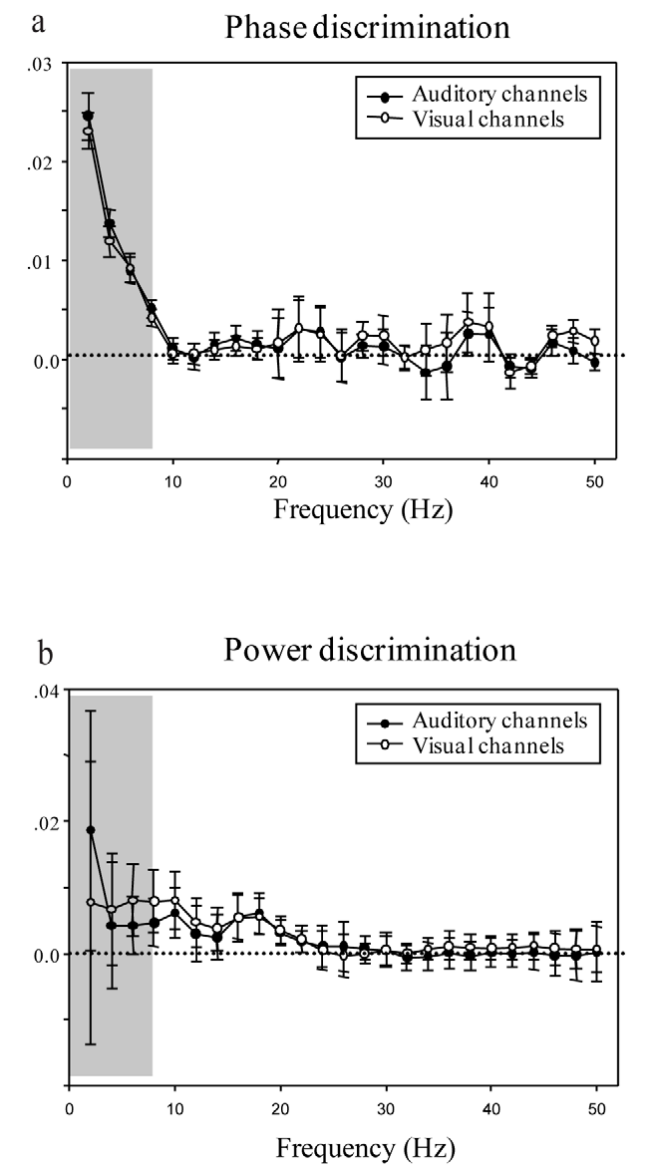
Phase-based and Power-based movie discrimination ability. Phase (a) and power (b) discrimination ability as a function of frequency (2–50 Hz) for 20 auditory (solid circles) and 20 visual channels (open circles) selected from separate auditory and visual localizer pretests for each participant. The gray box denotes the delta-theta range (2–7 Hz) selected for further analyses. The phase discrimination score in this range is significantly above 0. Error bars indicate the standard error across the 36 calculated samples (six stimulus conditions, six subjects).

We first assessed whether MEG responses in single trials can reliably track the six movie clips we presented to participants (three Matched, three Mixed). The phase and power pattern of MEG responses to the movies (see illustration of cross-trial phase coherence analysis in Figure 1a) and the corresponding discrimination ability were calculated as a function of frequency of the brain response (0–50 Hz) using previously developed methods [46]. We quantified stimulus-specific trial-by-trial phase and power pattern coherence in 20 auditory and 20 visual channels, which were defined in separate auditory (1 kHz tone pip) and visual (alternating checkerboard) localizer pretests for each subject (see Figure S2). As illustrated in Figure 2a, both auditory and visual cortical responses showed good discrimination ability in the delta-theta-band (2–7 Hz) phase pattern (above zero discrimination score, 2-way ANOVA, main effect of frequency, *F*(24, 840) = 7.94, *p*<0.0001; post-hoc one-sample *t* test in delta-theta band (2∼7 Hz), Auditory: *t* = 11.57, *df* = 35, *p*<0.0001, Visual: *t* = 11.16, *df* = 35, *p*<0.0001). Critically, phase tracking was not accompanied by comparable power pattern tracking (Figure 2b, 2-way ANOVA, main effect of frequency, *F*(24, 840) = 0.517, *p* = 0.97; *t* test in delta-theta band (2∼7 Hz), Auditory: *t* = 0.913, *p* = 0.368; Visual: *t* = 0.698, *p* = 0.49). These results demonstrate that the phase of ongoing auditory and visual cortical low-frequency oscillations is reliably modulated by the audio-visual stimuli, and thus conveys information about the rich naturalistic dynamics of these multi-sensory movies.

### Modality Specificity in Low-Frequency Phase Tracking

Having established the *sensitivity of the low-frequency phase pattern* to different audiovisual movie streams using the cross-trial phase coherence (Figure 1a), we next evaluated its *modality specificity* in auditory and visual cortical responses, by employing a cross-movie coherence analysis (Figure 1b; Figure S3 schematizes the logic). Given the predominantly unisensory characteristics of cortical responses early in the cortical processing hierarchy, the low-frequency phase pattern should be mainly driven by the stimulus in the corresponding sensory modality. We thus tested a double dissociation hypothesis, namely that in *auditory* channels, movie clips sharing the *same* auditory input regardless of visual input (stimuli we call “SameAud”) should induce a more similar low-frequency phase pattern response (and display higher cross-movie delta-theta phase coherence) than those containing the same visual but *different* auditory input (stimuli called “SameVis”); analogously, in *visual* channels, SameVis movies should yield higher cross-movie delta-theta phase coherence compared to SameAud movie pairs.

**Figure 3:**
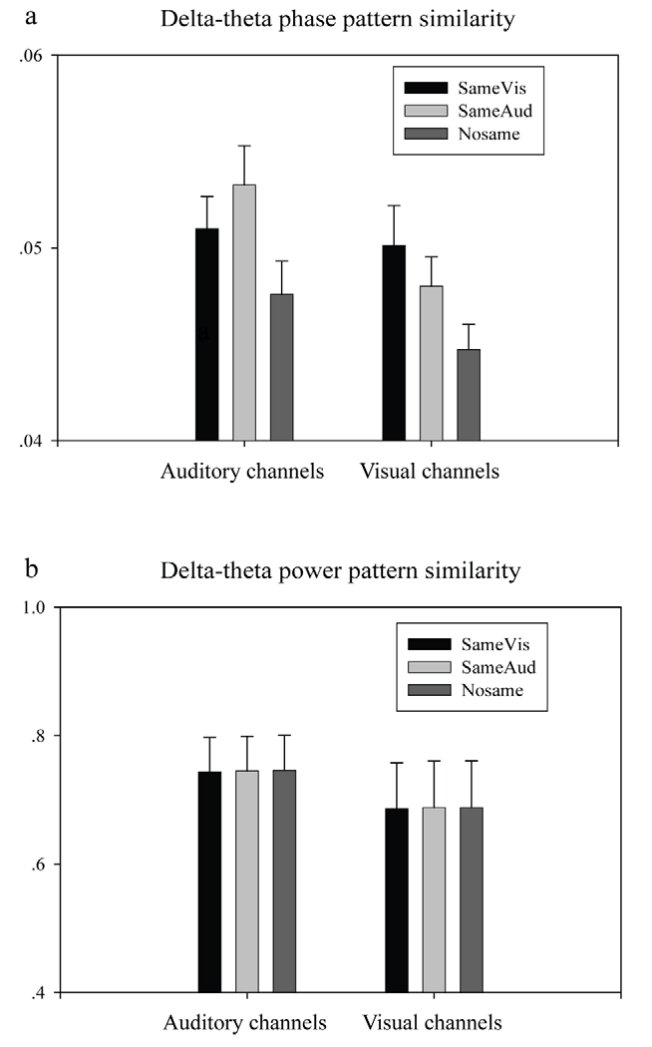
Low-frequency band phase pattern reflects within- and across-modality tracking. Cross-movie response coherence (how similar are the responses elicited by two movies) in delta-theta phase pattern (a) and power pattern (b) for the 20 auditory and 20 visual channels selected from independent localizer pretests (see Figure 1b and Methods for analysis illustration). SameVis: movie clip *pair* sharing the same visual but different auditory input; SameAud: movie *pair* sharing same auditory but different visual input; NoSame: movie pair differing in both auditory and visual inputs. For example, for movie clip V1A1, the SameVis, SameAud, and NoSame movies correspond to V1A3, V2A1, and V3A2, respectively. Error bars indicate the standard error across six subjects.

For the three matched clips (V1A1, V2A2, V3A3), we selected the corresponding SameVis and SameAud stimuli (see Figure 1b and Figure S3 for visualization of the design; e.g., for matched clip V1A1, its SameVis counterpart is V1A3, its SameAud is V2A1); we then calculated the similarity or coherence between the responses to matched clips and the corresponding SameAud or SameVis mixed clips (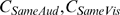), separately for auditory and visual areas. The cross-movie low-frequency phase coherence results (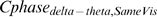, 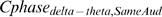) show a double dissociation (Figure 3a; condition×place interaction, *F*(1, 5) = 10.44, *p* = 0.023). This confirms the efficacy of the auditory and visual “functional channel localizers”; more importantly, though, this analysis suggests, plausibly, that the phase patterns over auditory and visual areas are predominantly driven by the sensory stimulus structure in the corresponding modality. Critically, the corresponding power coherence (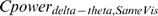, 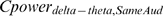) did not show the double dissociation pattern (Figure 3b; condition×place interaction, *F*(1, 5) = 0.077, *p* = 0.79), confirming that *precise timing*—as reflected in the phase of delta and theta activity—plays a dominant role in sensory stream representation.

**Figure 4:**
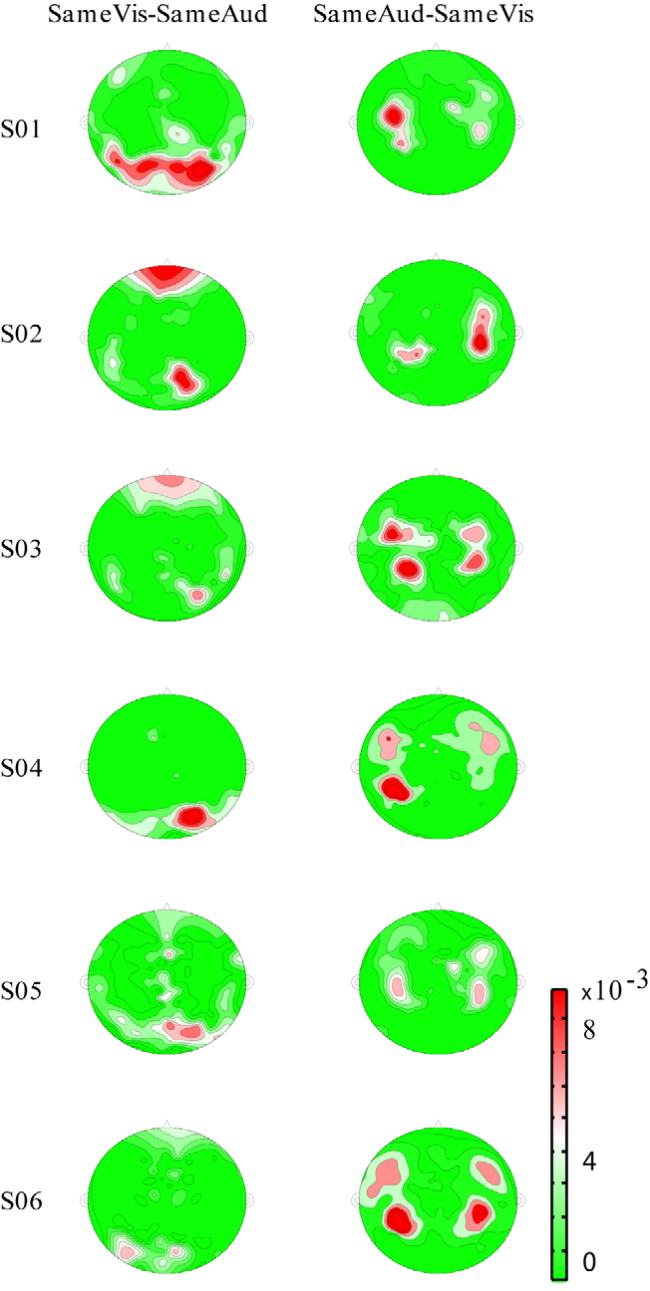
Low-frequency cross-movie phase coherence distribution map. Delta-theta cross-movie phase coherence distribution map for each of the six subjects, indicating within-modality tracking. In this flat map of the MEG recordings, left is left, right is right, and red indicates larger cross-movie phase coherence. Left: distribution map for larger cross-movie delta-theta phase response coherence of SameVis movie pair versus SameAud movie pair. The comparison implicates occipital (visual) cortex. Right: distribution map for larger cross-movie delta-theta phase coherence of SameAud movie pair versus SameVis movie pair. This analysis shows auditory activation.

The modality-*dependent* characteristics of the delta-theta phase pattern in all 157 recorded channels were verified by comparing the spatial distribution maps of the cross-movie delta-theta phase coherence (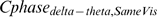, 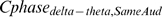). We observed a lateral temporal origin of 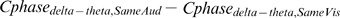 and an occipital origin of 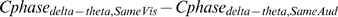 in every subject (Figure 4). The spatial distribution results thus confirm the finding that in response to a multi-sensory audiovisual stream, the low-frequency phase of the auditory and visual cortical activities principally and concurrently tracks the *respective* sensory stimulus dynamics.

### Cross-Modality Low-Frequency Phase Tracking

We then examined the critical hypothesized *cross-modality modulation effects* in the low-frequency phase pattern, by studying whether naturalistic visual input can affect the phase of auditory cortical oscillations (as previously only observed using artificial stimuli and in animal data), and similarly whether the auditory dynamic structure influences the phase of ongoing rhythmic activities in visual cortex, to some extent. A cross-movie coherence analysis was again performed (Figure 1b; Figure S3 schematizes the logic), by calculating the coherence or similarity between the responses to matched clips and the corresponding NoSame mixed clips, i.e. movie clip differing in both auditory and visual input (e.g., for matched clip V1A1, V2A2, V3A3, their respective NoSame counterpart is V3A2, V1A3, V2A1), in auditory and visual areas separately.

The logic of this analysis is as follows: If the low-frequency phase pattern in one sensory modality is systematically influenced by the other modality, movies sharing same visual input (SameVis) should show more similar low-frequency phase pattern in *auditory* cortex, compared to movies differing in both visual and auditory inputs (NoSame); similarly, in visual cortex, the SameAud movies should show higher cross-movie coherence than NoSame movies. Figure 3a shows that the NoSame pair manifested the smallest cross-movie phase coherence (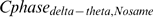), supporting our hypothesis (3-way ANOVA, condition main effect, *F*(2, 10) = 36.394, *p*<0.0001; post-hoc analysis, NoSame versus SameVis, *p*<0.0001, NoSame versus SameAud, *p*<0.0001; condition×place interaction, *F*(2, 10) = 8.467, *p* = 0.007). The delta-theta power pattern reflects no such effect (Figure 3b). This suggests that in response to an audio-visual stream (e.g., V1A1), the phase of the cortical activity is driven and modulated not only by the input in the corresponding modality (double dissociation result discussed above) but also by input from another modality (cross-sensory phase modulation).

### Matched Movies Elicit Stronger Trial-to-Trial Low-Frequency Phase Pattern

**Figure 5:**
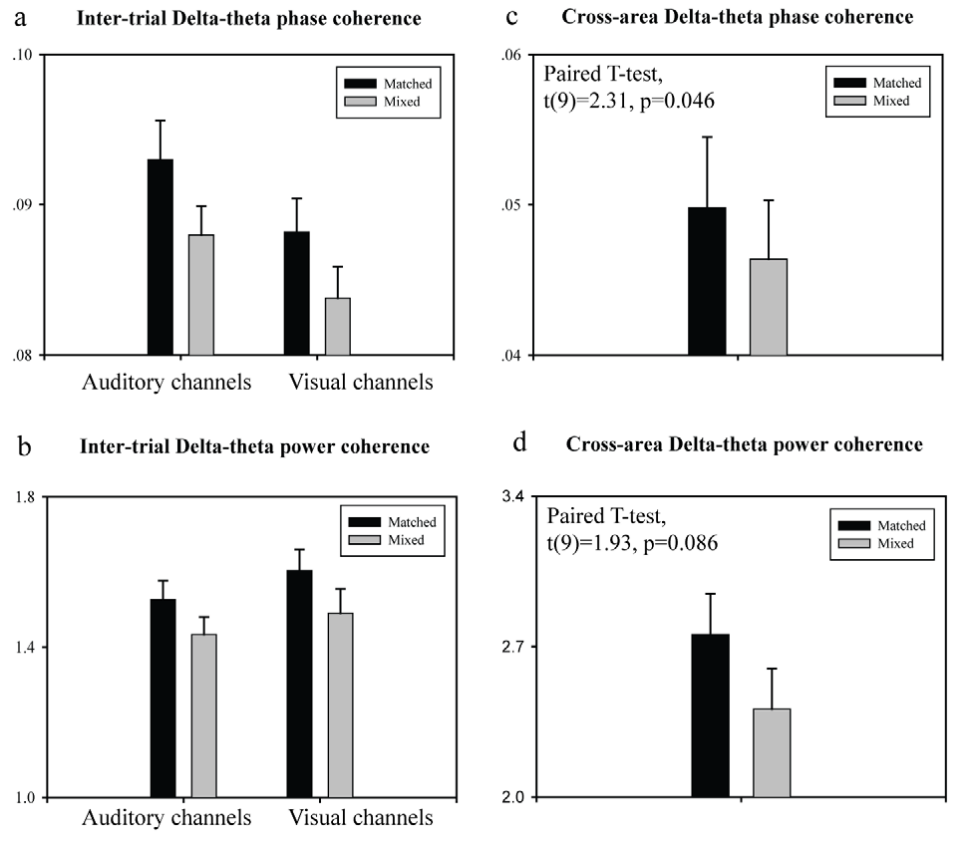
Inter-trial low-frequency phase coherence depends on audiovisual temporal. Cross-trial coherence in delta-theta phase pattern (a) and power pattern (b) for Matched (black bar) and Mixed (grey bar) stimulus conditions, in 20 auditory and 20 visual channels (see Figure 1a and Methods for analysis illustration). Cross-area (auditory and visual) coherence in delta-theta phase pattern (c) and power pattern (d) for Matched (black bar) and Mixed (grey bar) stimulus conditions. Error bars indicate the standard error across 10 subjects.

The above *cross-movie* coherence results demonstrate that the phase pattern in response to an audiovisual stream carries information about both auditory and visual stimulus structure. We next ask whether multisensory tracking is simply a mixture of passive following responses to unisensory stimuli, or—more interestingly—whether phase-tracking plays an *active* role in multisensory integration, by establishing a cross-modal temporal context in which a unisensory stimulus unfolds and merges into a coherent perceptual representation. We first examined the similarity in the elicited phase pattern response in auditory and visual areas. Given the congruent temporal structure in matched audiovisual stimuli, together with the observed within-modality phase tracking, we predict that both auditory and visual areas show higher similarity in low-frequency phase responses for the matched conditions. The cross-movie analysis results support the hypothesis (Figure 5c, paired *t* test, *t*(9) = 2.31, *p* = 0.046); the corresponding power coherence revealed no statistical difference (Figure 5d, paired *t* test, *t*(9) = 1.93, *p* = 0.086).

In light of the observed similarity between the phase response in the two modalities, we next conjecture that the cross-modality phase modulation will occur in a manner “temporally commensurate” to within-modality phase modulation, leading to more temporally reliable integration and consequently achieving a more *robust* low-frequency-based representation of audio-visual naturalistic stimuli (enhanced trial-to-trial response reliability) in both sensory areas (not between areas). Importantly, the *cross-trial reliability enhancement* hypothesis cannot be derived from a passive following response interpretation.

We compared the delta-theta cross-trial phase coherence for the three matched and three mixed movies separately, noting that the three movies in the mixed group contained exactly the same auditory and visual inputs as the matched one—but in incongruent audio-visual combinations (Figure 1a). We observed stronger trial-by-trial delta-theta phase pattern coherence in the matched group than in the mixed group (2-way ANOVA, significant main effect of condition, *F*(1, 9) = 7.33, *p* = 0.024), in both auditory and visual areas (Figure 5a). The cross-trial power coherence revealed no significant difference between the two conditions (Figure 5b, condition main effect, 2-way ANOVA, *F*(1, 9) = 3.64, *p* = 0.09). The result that the trial-by-trial phase reliability depends on the relative audiovisual temporal relationship thus supports the “*active cross-modal phase modulation*” hypothesis for multisensory integration. In our view, sensory cortical activity builds a more efficient and robust continuous representation for a temporally congruent multi-sensory stream by mutually modulating the low-frequency phase of ongoing oscillatory activity in an *active* manner, perhaps facilitating temporal packaging of information that can then act “predicatively” across modalities.

### Classification Based on Low-Frequency Phase Pattern

**Figure 6:**
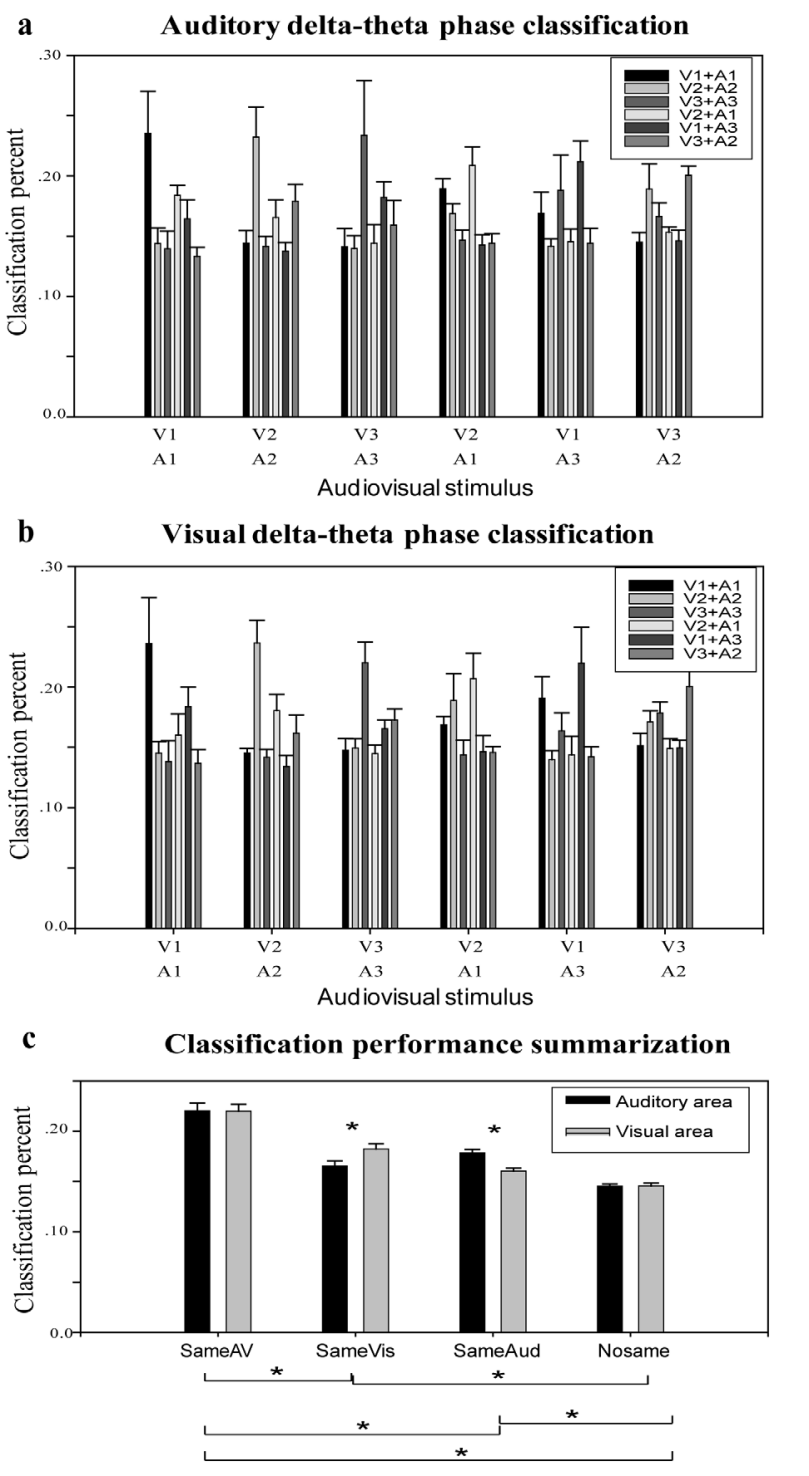
Low-frequency phase-pattern-based classification performance. Grand average of delta-theta-phase-based classification histograms for each of the six audiovisual stream conditions (3 matched and 3 mixed conditions) for auditory (a) and visual areas (b). Note that the sum of the clustered bar sums to 1. Error bars indicate the standard error across six subjects. (c) Generalization and statistical analysis of classification performance (ab).

To apply a unified analysis framework to our data, a classification analysis was employed based on the low-frequency (2–7 Hz) phase pattern in single response trials across all six movies. For each of the six movie clips, the delta-theta phase pattern as a function of time for one single trial response under one stimulus condition was arbitrarily chosen as a template response for that movie. The delta-theta phase pattern of the remaining trials of all stimulus conditions was calculated, and their similarity to each of the six templates was defined as the distance to the templates. Responses were then classified to the closest movie template. The classification was computed 100 times for each of the 20 auditory and 20 visual channels in each subject, by randomly choosing template combinations. This classifier analysis shows that the delta-theta phase pattern successfully discriminates among movies. The individual trial data for each condition were predominantly classified as belonging to that condition, for both auditory (Figure 6a) and visual (Figure 6b) areas. Second, the classification results support the tracking hypothesis for matched versus mixed conditions, revealing higher “self”-classification for matched than mixed movies. Third, the modality-specific characteristics of phase tracking were manifested in the classification in that in auditory areas, each of the six movies was categorized to the movie stimulus sharing the same auditory input (SameAud) with larger proportion than to SameVis input, and vice versa for visual areas. Finally, the classification results also support the elevated response reliability by congruent audiovisual stimuli. The response to each movie clip was primarily classified to itself, secondly to the clip sharing the same modality (e.g., SameAud for auditory channels), and thirdly to the movies sharing the same input in the other modality (e.g., SameVis in auditory area), which has a significantly better classification proportion than stimuli differing in both inputs (NoSame). A statistical analysis and summary of the classification data (Figure 6c) underscores the effect of this cross-sensory phase modulation. The results demonstrate that the low-frequency phase pattern in sensory cortices can be relied on for audiovisual stream discrimination in single trial responses, and that it is modulated by input from multiple sensory domains, reflecting an *active* cross-sensory integration, dynamically evolving in time.

### Optimal Phase and Active Cross-Modal Low-Frequency Phase Modulation

Neurophysiological work in animal preparations suggests that non-auditory inputs can modulate auditory responses towards a preferred excitability state, by aligning the phase of ongoing low-frequency auditory activity with a specific phase angle known to elicit maximal stimulus-driven responses, resulting in the cross-sensory response amplification [20],[22]. We hypothesize that stimulus-induced temporal regularization leads to robust phase tracking, by resetting the phase of the intrinsic low-frequency rhythmic activity to a preferred phase. We thus expect (i) that the cross-trial delta-theta phase coherence is phase dependent, and the phase values corresponding to high cross-trial phase coherence values are non-uniformly distributed and centered on a preferred phase angle, and (ii) that the matched movie elicits a larger fraction of optimal phase compared to the mixed condition, since a temporally congruent stream would achieve cross-sensory phase tracking enhancement, by regularizing low-frequency phase to the optimal phase angle more robustly in each response trial.

**Figure 7:**
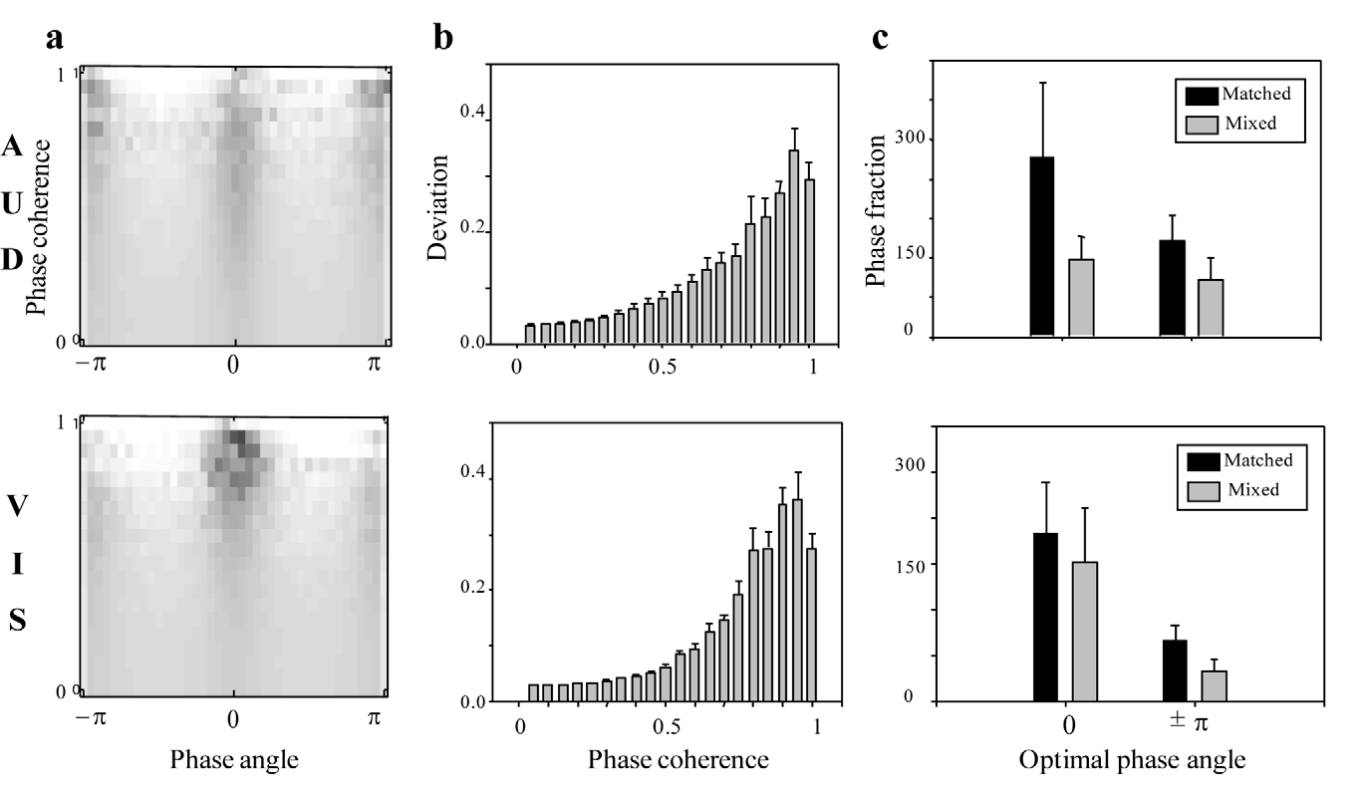
Low-frequency phase coherence and “optimal phase.” (a) Grand average of phase histograms (*x*-axis) as a function of inter-trial delta-theta phase coherence (*y*-axis, 0∼1) across six subjects in auditory channels (upper) and visual channels (lower). Note that the sum of each row is 1. (b) Deviation score from uniform distribution as a function of inter-trial delta-theta phase coherence (*x*-axis, 0–1). Error bars indicate the standard error across subjects. (c) “Optimal” phase (0 and ±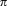) fraction for matched (black bar) versus mixed (grey bar) conditions in auditory (upper) and visual (lower) channels. Error bars indicate the standard error across six subjects.

We explored the relationship between the cross-trial phase coherence and the corresponding phase angles and observed an increasingly clustered phase angle distribution (around 0 and ±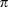) for higher phase coherence in both auditory and visual areas (Figure 7a, upper and lower panel). As shown in Figure 7b, we further quantified the deviation of phase distribution from uniform distribution as a function of cross-trial phase coherence values, and the results confirm that higher phase coherence corresponds to larger deviation from uniform distribution (2-way ANOVA, *F*(19, 95) = 67.99, *p*<0.001), thus suggesting a trend of non-uniform phase clustering for the robust phase tracking pattern. (Note that the drop in the deviation values for the highest phase coherence (∼1) may be due to the artifacts produced by small samples and large variance across subjects during such a high coherence regime.) The findings demonstrate that it is mainly the stimulus-induced delta-theta phase resetting to the preferred phase angle (0 or ±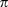) that regularizes the low-frequency phase pattern in each response trial to improve the phase tracking reliability. In addition, as shown in Figure 7c, the matched movies showed a larger fraction of optimal phase angle (0 or ±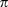) than mixed movies for higher phase coherence (>0.7) in both auditory and visual areas, as hypothesized; statistical testing confirms that phase angle at ±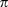 was more relevant to preferred or optimal phase (2-way ANOVA, main effect of condition, *F*(1, 5) = 5.794, *p* = 0.06) than phase angle at 0 (2-way ANOVA, main effect of condition, *F*(1, 5) = 2.856, *p* = 0.152), commensurate with optimal phase findings in neurophysiological studies [20],[22],[45]. The results support the view that the visual (auditory) stream in a matched movie modulates the auditory (visual) cortical activity by aligning the phase to the optimal phase angle so that the expected auditory (visual) input arrives during a high excitability state, to be amplified and achieve the cross-sensory enhancement. In contrast, mixed, incongruent audiovisual streams cannot benefit from the cross-sensory phase regularization and thus are driven to the preferred phase angle with a significantly smaller fraction than matched movie stimuli.

## Discussion

We examined multi-sensory interaction in early sensory areas in MEG responses recorded from human subjects viewing and listening to natural audio-visual movies. We show that the low-frequency, delta and theta phase pattern in early visual and auditory cortices tracks (and can discriminate among) naturalistic visual and auditory stimuli, respectively, in single MEG response trials. In addition, the low-frequency phase pattern in one sensory domain can, to some extent, represent and track the stimulus structure of the other modality. Importantly, temporally aligned audio-visual streams (“matched”) elicit stronger low-frequency trial-by-trial phase response reliability than non-aligned streams (“mixed”), supporting an active cross-modal phase modulation versus a “passive stimulus following response” interpretation. Finally, the delta-theta phase clusters for stronger phase tracking, indicating that it is phase resetting to the preferred or “optimal phase” that tracks the “within-modality” and “across-modality” stimulus structure. Congruent multisensory stimuli lead to mutual driving towards “optimal phase” more reliably, perhaps to achieve temporally optimized cross-sensory enhancement. We conjecture that the ongoing phase pattern of slow oscillatory activity in sensory cortices provides a unified temporal frame of reference in which continuous multi-sensory streams are seamlessly represented and integrated into a coherent percept.

### Phase Tracking of Naturalistic Sensory Streams

Unlike pairings of transient artificial stimuli used in most previous audiovisual studies, we examined the cross-modal integration effects in presumptively unimodal areas by employing naturalistic audiovisual movies that are ethologically natural and extended in time (30-s film clips). Naturalistic stimuli contain complex structure and rich dynamics in the time domain, and it has been suggested that the relevant neural mechanisms are in part shaped by the statistical structure of natural environments [47],[48]. Our previous MEG studies revealed that the phase pattern of theta-band responses reliably tracks and discriminates natural spoken sentences [46]. Here we build on and extend the previous findings by showing that delta-theta phase tracking exists for multi-sensory streams and that the low-frequency phase response in auditory *and* visual cortices reliably tracks audio-visual movies concurrently. There is emerging consensus that the signals quantified in neuroimaging (e.g., MEG signals) reflect synchronized large-scale neuronal ensemble activity and have been found to mainly derive from LFP rather than spiking activity [49]. A recent neurophysiological study in monkeys quantified the information different codes carry about natural sounds in auditory cortex and found that spiking responses interpreted with regard to the relative phase of the accompanying slow ongoing LFP are more informative about the properties of the dynamic sound than spiking responses alone [50]. The same encoding scheme has also been observed in visual cortex in response to natural movies [51]. Our results from human neuroimaging converge with these neurophysiological studies on low-frequency phase tracking for naturalistic streams and are commensurate with the observed essential role of brain oscillations in sensory processing, feature integration, and response selection within the various sensory modalities [30],[34]–[36],[52]. It has been argued that intrinsic rhythms undergo significant phase resetting in response to stimulus presentation [35],[53],[54], and crucially, some studies demonstrate that neuronal oscillations enhance the response robustness to natural stimulation by modulating the excitability state (phase resetting) for spiking activity [55].

### Phase Tracking and Attention

Could one argue that the observed delta-theta phase tracking is due to different levels of attention to a given modality, given the important role of attention in multisensory integration [25],[56],[57]? Such a view cannot be a sufficient explanation because the low-frequency phase pattern distinguishes the audio-visual streams belonging to the matched *or* mixed conditions, both of which elicit similar attentional states. (The three matched (or mixed) movies should elicit similar attentional states, and therefore the delta-theta phase pattern should not be able to discriminate them only based on attentional state.) Interestingly, previous studies show that such cross-sensory interactions occur in anaesthetized animals [19],[21]. These observations suggest that the general attentional level is not the main source underlying the observed delta-theta phase tracking. Recent studies [56],[57] revealed that the phase of low-frequency oscillations in auditory and visual cortex entrains to the rhythm of the attended sensory stream amidst multi-sensory inputs and thus could track either a visual or auditory stimulus. They suggest the phase modulation mechanism to underlie temporally based attention. Their results further challenge an attentional-load explanation for the present data, given the observed modality-specific characteristics (the double dissociation results), and support that the observed delta-theta phase tracking is not due to global modality-independent attentional modulation.

Uncontrolled eye movements also constitute a possible confounding factor, given previous findings reporting the effect of eye position on the auditory cortical responses [17]. We believe that the eye-movement-related activity *may* contribute to phase modulation in early sensory activity, but not in a dominant way, given that the cross-modal phase modulation exists under both anesthetized conditions [19],[21] and controlled eye fixation conditions [22]. Note that eye movements by themselves cannot account for the observed stronger modulation for matched over mixed audiovisual stimuli; both carry the *same* visual stream; which should result in a comparable pattern of eye movements. More generally, during the free viewing of movies, eye movements are argued to be tightly correlated with stimulus dynamics, which in turn induces phase tracking in brain signals, and therefore the phase modulation mechanism may also be integral to the temporally based attention. Fries [35] recently proposed a rhythmic input gain model to link attention to brain oscillations and suggested that the strength of gamma-band synchronization (binding by synchronization) is modulated with the theta rhythm, the phase of which makes or breaks selections of input segments, thus constituting a strong link to the “biased competition” modal in visual attention [33].

### Temporal Scales, Brain Oscillations, and Natural Statistics

We found that low-frequency phase patterns were sufficiently reliable to continuously track the naturalistic audiovisual streams. The crucial relevance of low-frequency oscillations to perceptual analysis has been observed in several studies [20],[22],[46],[50],[51]. The acoustic structure of both natural sounds and movies contain rich dynamics on multiple time scales, but with power dominance in the low-frequency range [48],[58]–[60]. Accumulating evidence demonstrates that a coarse representation suffices for the comprehension of natural streams [61]. For example, from the perspective of speech processing, a temporal window of ∼200 ms corresponds to mean syllable length across languages, and such a temporal window has been suggested as a fundamental unit for speech perception [62],[63]. The observed tracking ability of slow quasi-rhythmic (and aperiodic) activity may be simply driven by the input temporal pattern, but we conjecture that it reflects an internal stable processing rhythm [64] that is ideally suited to match the gross statistical temporal structure of natural streams. Recent data [65] demonstrate robust temporal correspondence in the delta-theta range (2∼7 Hz) between visual and auditory streams in multisensory speech signals, supporting this interpretation.

In addition to the essential role of long-duration time scales in natural stimuli, the dynamic structure at other biologically relevant scales, especially the short windows (e.g., ∼25 ms) corresponding to gamma band oscillation, also carries important information [62],[64]. Several previous studies show the relevance of gamma oscillations to multisensory integration, but in contexts of transient or evoked responses [40],[42], which is a very different approach from ours. In the current work, we examine the sustained response pattern to natural complex audiovisual scenes and the relevance to multisensory integration. A possible factor accounting for the absence of evidence for fast, gamma rhythms in tracking might lie in the task demands; subjects were only asked to passively view and listen to the audiovisual streams, without requiring their focused, selective attention to fast transitions, phonemes, any aspect of sublexical information, etc. Crucially, both unimodal and multimodal naturalistic streams contain various temporal scales that are nested within each other. For example, in human speech, high-frequency events (e.g., formant transitions) are temporally nested within low-frequency structures (e.g., syllables, phrases). Correspondingly, human cortical oscillations at different frequencies also manifest similar temporally nested relationships and tend to be phase-amplitude coupled [66]. Such cross-scale coupling in both naturalistic extended stimuli and brain oscillations are consistent with the “sampling window hypothesis” for speech perception [62], and further indicate a general cross-scale modulation mechanism underlying multi-sensory interaction [56].

### Phase-Reset Mechanisms and Active Multisensory Interaction

The central finding concerns the hypothesis of active cross-modality phase modulation of endogenous oscillations in a multi-sensory context. Specifically, we observed that the auditory and visual modalities can mutually and actively modulate the phase of the internal low-frequency rhythms in early sensory cortical regions and that such cross-sensory driving efficiency depends on the relative audiovisual timing. A study recording A1 in awake macaques [20] revealed phase modulation in multi-sensory interaction: somatosensory inputs enhanced auditory processing by resetting the phase of ongoing neuronal oscillations in A1 so that the accompanying auditory input arrived during a high-excitability phase. A further neurophysiological experiment exploring the impact of visual stimulation on auditory responses demonstrated that visual stimuli modulated auditory cortex activity, at the level of both LFP and single-unit responses [22]. Importantly, they too found that the observed cross-sensory enhancement correlated well with the resetting of slow oscillations to an optimal phase angle, and the multi-sensory interactions were sensitive to the audiovisual timing. Moreover, they discovered that matched audiovisual stimuli enhanced the trial-to-trial response reliability in auditory cortex of alert monkeys [45], precisely like one of our central findings of a tight link between cross-sensory modulation efficacy and relative audiovisual timing congruency. Our results in humans are thus in good agreement with these animal data and also implicate neural mechanisms accounting for previous behavioral results showing temporally matched visual amplification of auditory processing, in both monkeys [67] and human subjects [4],[68].

Given the simple binary design here (matched versus mixed), further studies need to be executed by continuously jittering the temporal relationship between auditory and visual stimuli and investigating the influences in both behavior and cross-modal low-frequency phase modulation in a more systematic way. Recently, Schroeder et al. [44] proposed a phase-resetting-based mechanism to solve the “cocktail party” problem using such a mechanism and hypothesized that the visual amplification of speech perception is operating through efficient modulation or “shaping” of ongoing neuronal oscillations. Our results support such a model and indicate that multi-sensory integration is at least in part based on a cross-modal phase resetting mechanism in early cortical sensory regions. The phase patterns of the ongoing rhythmic activity in early sensory areas help construct a temporal framework that reflects both unimodal information and multimodal context from which the unified multisensory perception is actively constructed. However, we do not exclude the existence of multiple multisensory integration pathways, as shown in a recent study [29] demonstrating the convergence of lateral and feedback in multisensory integration, given the complex characteristics of integration. In a more general sense, we surmise that the dynamic interplay of neural populations [28] constitutes a unified temporal framework where the segmented senses unfold and merge, resulting in the seamless multisensory-integrated dynamic world we perceive. Further human studies with better spatial resolution (e.g., intracranial EEG in humans and fMRI+EEG recording) may help to address the issue in a more granular way. The results from this human MEG experiment suggest that neuroimaging data can make a fruitful contribution to our understanding of neural coding, building on concepts of neural timing that can be exploited productively at the levels of analysis of large neuronal populations.

## Materials and Methods

### Subjects and MEG Data Acquisition

Six right-handed subjects provided informed consent before participating in the experiment. All subjects had normal vision and hearing. We have acquired data from additional four subjects (10 subjects in total then) to specifically investigate matched versus mixed cross-trial low-frequency phase coherence difference (as shown in Figure 5). Neuromagnetic signals were recorded continuously with a 157 channel whole-head MEG system (5 cm baseline axial gradiometer SQUID-based sensors; KIT, Kanazawa, Japan) in a magnetically shielded room, using a sampling rate of 1,000 Hz and an online 100 Hz analog low-pass filter, with no high-pass filtering.

### Stimuli and Experimental Procedures

Three audio-visual movie clips (V1+A1, V2+A2, V3+A3) were selected from the movie “Dumb and Dumber” (1994, New Line Platinum Series) to form the three “Matched” movie stimuli (see Figure S1). We constructed another three “Mixed” movie clips, by shuffling the auditory and visual combinations (V1+A3, V2+A1, V3+A2). All six movie clips contained natural conversation in an audiovisual setting and were 30 s in duration. Prior to the movie experiment, the subjects participated in one auditory localizer pretest in which they were presented with 1 kHz tone pips (duration 50 ms) and one visual localizer pretest in which they were presented with alternating checkerboard stimuli. Both pretests were performed to collect functional localization data for auditory and visual cortices (to identify the most responsive channels, Figure S2). Subjects were told to passively view and listen to the six audio-visual stimulus streams (no explicit task) presented on a rear projection screen in the shielded room screen (the clips subtended ∼18 deg horizontal and 11 deg vertical visual angles, presented at typical photopic luminance values) without restriction on eye movements. Each of the six movie clips was presented 15 times, in two separate blocks (Matched block and Mixed block), with the audio track presented at a comfortable loudness level (∼70 dB).

### Data Analysis

In the auditory localizer pretest, the large electrophysiological response peak with latency around 100 ms after tone-pip onset was determined (M100 or N1m) and the 20 channels with largest response amplitude were defined as the *auditory channels*. These channels, unsurprisingly, largely lie over the temporal lobe. In the visual localizer pretest, the 20 channels with largest response amplitude at the response peak with latency around 150 ms were selected as *visual channels* (typically occipital). The channel selection procedure was performed for each subject separately, and all subsequent analysis was done on those independently selected channels to represent auditory and visual cortical activity, respectively. There was no overlap among the channel groups.

For each of the six audio-visual stimuli (15 trials of each), 12 out of 15 response trials were chosen and termed “within-group” signals (six within-group signals corresponding to six movie stimuli). Note that selecting 12 trials out of 15 trials here was simply due to this specific discrimination analysis that required trial number to be an integer number of 6 (the stimulus condition number); the following other analyses were performed on all the 15 response trials. Two response trials (one-sixth of the 12 trials for each stimulus condition) were chosen from each of the six groups and combined to construct a 12-trial “across-group” signal. Six across-group signals were constructed by repeating the combination procedure six times. For each of the twelve 12-trial signal groups (six *within-group* and six *across-group* signals), the spectrogram of the entire 30 s of each single trial response was calculated using a 500 ms time window in steps of 100 ms, for each of the 20 auditory channels and 20 visual channels defined for each subject. The phase and power were calculated as a function of frequency and time and were stored for further analysis. The “cross-trial phase coherence” (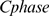) and “cross-trial power coherence” (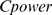) were calculated as
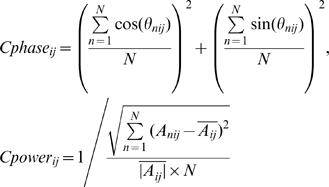
where 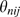 and 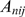 are the phase and absolute amplitude at the frequency bin i and temporal bin j in trial n, respectively. These calculated cross-trial coherence parameters (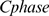 and 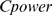) are dimensionless quantity and were compared between each of six within-group signals and each of six across-group signals separately. The discrimination function (also dimensionless quantity) for each frequency bin i was defined as
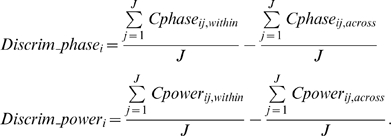



The resulting six discrimination functions for each of the six subjects were then averaged. A value significantly above 0 indicates larger cross-trial coherence of within-group signals than across-group signals. The average values within delta and delta-theta ranges (∼2–7 Hz) from 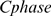 and 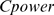 were then selected for further analysis, given the above-zero discrimination score in this frequency range in 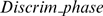 function (upper panel of Figure 1). Importantly, note the different formulas from which phase coherence and power difference are derived, due to their different characteristics. We calculated power coherence in terms of the cross-trial standard deviation of power pattern normalized by the power in each frequency band, similar to the Fano factor calculation in neurophysiology, but the value is in reversed direction (smaller Fano factor corresponds to larger reliability, and Fano factor can be below or above 1). Therefore, correspondingly, the power coherence values, as a result of the current computation, would not necessarily be smaller than 1, which is different from the phase coherence range (0–1), and therefore cannot be directly compared as quantities.

For the *cross-movie coherence analysis* (Figure 3, Figure 4), for each of the three matched movie clips (V1A1, V2A2, V3A3), we first selected the corresponding SameVis (V1A3, V2A1, V3A2), SameAud (V2A1, V3A2, V1A3), and NoSame (V3A2, V1A3, V2A1) movie stimulus in the mixed group, and then calculated the cross-movie delta-theta phase coherence (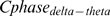) and power coherence (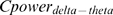) (both of them are dimensionless quantities) for each of the 20 auditory and 20 visual channels defined in localizer pretest in each subject, by
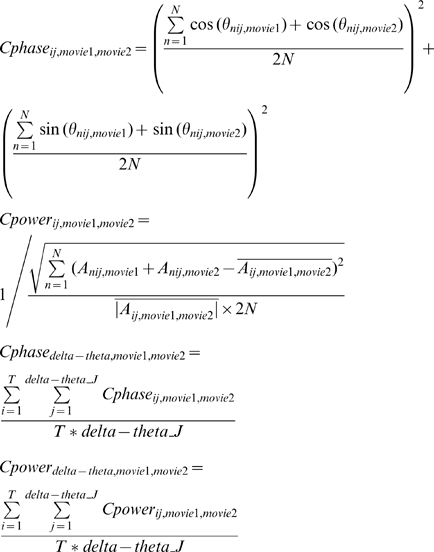



Note that the cross-movie coherence values derived from the above equation actually quantify the similarity extent of the response from two movies, in either phase or in power pattern (see Text S1 for the difference between the cross-movie analysis employed here and traditional cross-channel coherence analysis). For example, 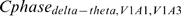, 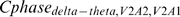, and 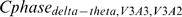 indicate how similar the delta-theta phase responses elicited by two movies sharing the same visual stream but different auditory input are (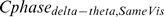, as shown in Figure 3). We calculated it in auditory channels and visual channels separately.

The across-movie delta-theta phase coherence distribution maps (Figure 4) for 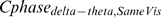 and 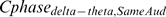 conditions were constructed, respectively, in terms of the corresponding values of all 157 MEG channels for each subject.

To evaluate the low-frequency *inter-trial phase and power coherence* (Figure 5ab) for matched (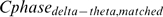, 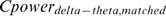) and mixed (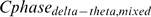, 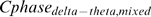) conditions, we first calculated the low-frequency inter-trial phase coherence for each of the six movie stimuli (Movie1∼Movie6: V1A1, V2A2, V3A3, V1A3, V2A1, V3A2) and then averaged the inter-trial delta-theta phase coherence and power coherence for the three matched movies and the three mixed movies separately, by
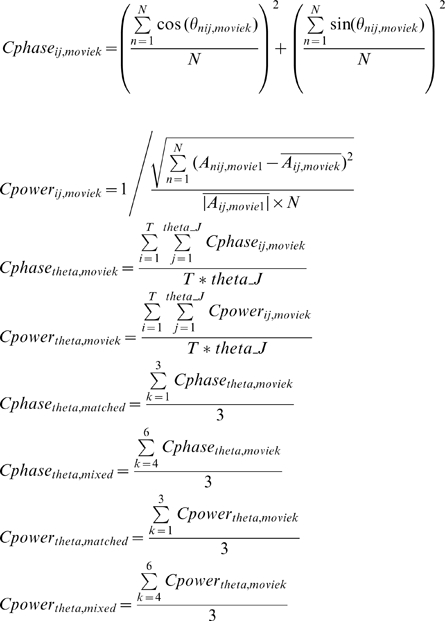



The cross-area analysis is similar to the cross-movie analysis but calculates the pattern similarity between auditory channels and visual channels, instead of that between movie 1 and movie 2 in auditory and visual channels separately in cross-movie analysis.

In the classification analysis (Figure 6), for each of the six movies, the delta-theta phase pattern as a function of time for one single trial under one stimulus condition was arbitrarily chosen as a template response for that movie. The delta-theta phase pattern of the remaining trials of all stimulus conditions was calculated, and their similarity to each of the six templates was defined as the distance to the templates [46]. Responses were then classified to the closest movie template. The classification was computed 100 times for each of the 20 auditory and 20 visual channels in each subject, by randomly choosing template combinations.

In the optimal phase analysis (Figure 7), for each of the 20 auditory and 20 visual channels in each subject, the calculated cross-trial phase coherence 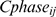 (i denotes time index and j denotes frequency index in range between 2∼7 Hz) was divided into 20 bins ranging from 0 to 1. The phase angle 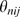 (n denotes the trial index) histograms in the range of 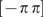 in each of the 20 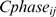 value ranges was then constructed, and the resulting matrix was averaged across six stimulus conditions and 20 selected channels for each subject (Figure 6a shows the grand average of the matrices). The deviation of the phase histogram 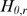 (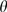 indicates the 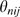 and 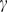 indicates the 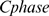) from uniform distribution 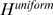 was quantified by deviation function as a function of 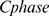 by 
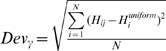
, as shown in Figure 6b.

We then selected all the phase angles with corresponding 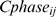 above 0.7 for all the selected channels in each subject and quantified the number of phase angles around 0 and around 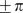 for the matched and mixed movie stimuli, respectively.

We also performed a control analysis to rule out “leaking” induced cross-modal modulation (see Text S2 for details).

## Supporting Information

Figure S1 **Audiovisual movie stimulus illustration.** Three matched audiovisual movie clip illustration (V1+A1, V2+A2, V3+A3). The three mixed audiovisual movie stimuli are mixtures of V2+A1, V1+A3, and V3+A2.
(0.67 MB DOC)

Figure S2 **Channel localization (linked to** **Figures 2** **,** **3** **,** **4** **,** **5** **,** **6** **,** **7** **in auditory and visual channels analysis).** Auditory and visual localizer-based contour map for one representative subject. Red indicates a large absolute response value around the M100 peak latency (auditory localizer) and the M150 peak latency (visual localizer). Of the 157 recorded channels, 20 auditory and 20 visual channels were chosen based on the contour map for each subject—with no overlap allowed (i.e., the main analyses are based on spatially distinct sets of channels). Predictably, the visual localizer implicates occipital channels (both on the left and right of the midline), and the auditory localizer reflects the more anterior canonical (dipolar) distribution that has two channel groupings around a temporal lobe source (M100 dipole pattern). The color bar is in units of fT.
(0.16 MB DOC)

Figure S3
**Cross-movie coherence analysis illustration (linked to** **Figure 3** **and** **Figure 6** **).** Illustration of the logic of cross-movie phase coherence analysis. In each of the six movie stimuli (first row of Figure S2), the solid bar represents the auditory stream and the hatched bar of the same color represents the corresponding visual stream. The middle and lower rows of Figure S2 indicate the hypothesized “representation ratio” of the stimulus in auditory and visual areas, respectively, in that the auditory stimulus dynamics will be more strongly represented in auditory cortex (solid bar) and the visual information (hatched bar) will be better represented in visual cortex. Crucially, if there exists direct modulation across sensory areas, the auditory area will *also* represent visual information, although to a lesser degree, and vice versa in the visual area. The figure illustrates an arbitrary hypothesized “representation distance” among the six movie stimuli in auditory and visual areas given the representation ratios in 2a. In this visualization, the distance between any two items corresponds to the similarity of the representation of the two movies, indicated by the arrow length between them (shorter distance means higher degree of similarity). D1, D2, and D3 correspond to the representation distance between one specific stimulus in the Matched group (A1V1 stimulus, for example) and the corresponding SameAud (A1V2), SameVis (A3V1), and NoSame (A2V3) counterparts in the Mixed group, respectively. A cross-modal representation results in the D2<D3 prediction for the auditory area and the D1<D3 prediction in the visual area. For example, the additional representation of visual information (hatched bar) in the auditory area makes the SameVis pair representation (D2) more similar (they both contain the representation for the same movie) compared to the NoSame pair. In contrast, as shown in Figure S2c, if there is no significant cross-modal representation (either no or an ineffective visual representation in auditory area and vice versa in the visual area), there will be not much difference in the distance for the SameVis pair and the NoSame pair (similar D2 and D3) in auditory areas, and similarly D1 and D3 in visual areas. Therefore, in summary, by comparing whether 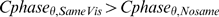 (D2<D3) in auditory channels and whether 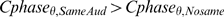 (D1<D3) in visual channels, we can examine and quantify the *cross-modal phase modulation* effect.(0.70 MB DOC)

Text S1 **Cross-trial phase coherence versus traditional coherence analysis.** Clarifying our cross-movie analysis, in comparison to traditional cross-channel coherence analysis.
(0.03 MB DOC)

Text S2 **Ruling out “leaking” induced cross-modal modulation.** Control analysis.
(0.03 MB DOC)

## References

[1] McGurk H, MacDonald J (1976). Hearing lips and seeing voices.. Nature.

[2] Driver J, Spence C (1998). Crossmodal attention.. Curr Opin Neurobiol.

[3] Shams L, Kamitani Y, Shimojo S (2000). Illusions. What you see is what you hear.. Nature.

[4] van Wassenhove V, Grant K. W, Poeppel D (2005). Visual speech speeds up the neural processing of auditory speech.. Proc Natl Acad Sci U S A.

[5] Jones E. G, Powell T. P (1970). An anatomical study of converging sensory pathways within the cerebral cortex of the monkey.. Brain.

[6] Linden J. F, Grunewald A, Andersen R. A (1999). Responses to auditory stimuli in macaque lateral intraparietal area. II. Behavioral modulation.. J Neurophysiol.

[7] Fuster J. M, Bodner M, Kroger J. K (2000). Cross-modal and cross-temporal association in neurons of frontal cortex.. Nature.

[8] Macaluso E, Driver J (2005). Multisensory spatial interactions: a window onto functional integration in the human brain.. Trends Neurosci.

[9] Calvert G. A (2001). Crossmodal processing in the human brain: insights from functional neuroimaging studies.. Cereb Cortex.

[10] Beauchamp M. S (2005). See me, hear me, touch me: multisensory integration in lateral occipital-temporal cortex.. Curr Opin Neurobiol.

[11] Ghazanfar A. A, Schroeder C. E (2006). Is neocortex essentially multisensory?. Trends Cogn Sci.

[12] Stein B. E, Stanford T. R (2008). Multisensory integration: current issues from the perspective of the single neuron.. Nat Rev Neurosci.

[13] Schroeder C. E, Foxe J (2005). Multisensory contributions to low-level, ‘unisensory’ processing.. Curr Opin Neurobiol.

[14] Calvert G. A, Bullmore E. T, Brammer M. J, Campbell R, Williams S. C (1997). Activation of auditory cortex during silent lipreading.. Science.

[15] Foxe J. J, Morocz I. A, Murray M. M, Higgins B. A, Javitt D. C (2000). Multisensory auditory-somatosensory interactions in early cortical processing revealed by high-density electrical mapping.. Brain Res Cogn Brain Res.

[16] Foxe J. J, Wylie G. R, Martinez A, Schroeder C. E, Javitt D. C (2002). Auditory-somatosensory multisensory processing in auditory association cortex: an fMRI study.. J Neurophysiol.

[17] Fu K. M, Johnston T. A, Shah A. S, Arnold L, Smiley J (2003). Auditory cortical neurons respond to somatosensory stimulation.. J Neurosci.

[18] Ghazanfar A. A, Maier J. X, Hoffman K. L, Logothetis N. K (2005). Multisensory integration of dynamic faces and voices in rhesus monkey auditory cortex.. J Neurosci.

[19] Bizley J. K, Nodal F. R, Bajo V. M, Nelken I, King A. J (2007). Physiological and anatomical evidence for multisensory interactions in auditory cortex.. Cereb Cortex.

[20] Lakatos P, Chen C. M, O'Connell M. N, Mills A, Schroeder C. E (2007). Neuronal oscillations and multisensory interaction in primary auditory cortex.. Neuron.

[21] Kayser C, Petkov C. I, Augath M, Logothetis N. K (2005). Integration of touch and sound in auditory cortex.. Neuron.

[22] Kayser C, Petkov C. I, Logothetis N. K (2008). Visual modulation of neurons in auditory cortex.. Cereb Cortex.

[23] Fuhrmann Alpert G, Hein G, Tsai N, Naumer M. J, Knight R. T (2008). Temporal characteristics of audiovisual information processing.. J Neurosci.

[24] Morrell F (1972). Visual system's view of acoustic space.. Nature.

[25] Macaluso E, Frith C. D, Driver J (2000). Modulation of human visual cortex by crossmodal spatial attention.. Science.

[26] Falchier A, Clavagnier S, Barone P, Kennedy H (2002). Anatomical evidence of multimodal integration in primate striate cortex.. J Neurosci.

[27] Rockland K. S, Ojima H (2003). Multisensory convergence in calcarine visual areas in macaque monkey.. Int J Psychophysiol.

[28] Senkowski D, Schneider T. R, Foxe J. J, Engel A. K (2008). Crossmodal binding through neural coherence: implications for multisensory processing.. Trends Neurosci.

[29] Arnal L. H, Morillon B, Kell C. A, Giraud A. L (2009). Dural neural routing of visual facilitation in speech processing.. Journal of Neuroscience.

[30] Singer W, Gray C. M (1995). Visual feature integration and the temporal correlation hypothesis.. Annu Rev Neurosci.

[31] Engel A. K, Fries P, Singer W (2001). Dynamic predictions: oscillations and synchrony in top-down processing.. Nat Rev Neurosci.

[32] Maier J. X, Chandrasekaran C, Ghazanfar A. A (2008). Integration of bimodal looming signals through neuronal coherence in the temporal lobe.. Curr Biol.

[33] Desimone R, Duncan J (1995). Neural mechanisms of selective visual attention.. Annu Rev Neurosci.

[34] Fries P (2005). A mechanism for cognitive dynamics: neuronal communication through neuronal coherence.. Trends Cogn Sci.

[35] Fries P (2009). Neuronal gamma-band synchronization as a fundamental process in cortical computation.. Annu Rev Neurosci.

[36] Jensen O, Kaiser J, Lachaux J. P (2007). Human gamma-frequency oscillations associated with attention and memory.. Trends Neurosci.

[37] von Stein A, Rappelsberger P, Sarnthein J, Petsche H (1999). Synchronization between temporal and parietal cortex during multimodal object processing in man..

[38] Sakowitz O. W, Quian Quiroga R, Schurmann M, Basar E (2005). Spatio-temporal frequency characteristics of intersensory components in audiovisually evoked potentials.. Brain Res Cogn Brain Res.

[39] Senkowski D, Molholm S, Gomez-Ramirez M, Foxe J. J (2006). Oscillatory beta activity predicts response speed during a multisensory audiovisual reaction time task: a high-density electrical mapping study.. Cereb Cortex.

[40] Senkowski D, Talsma D, Grigutsch M, Herrmann C. S, Woldorff M. G (2007). Good times for multisensory integration: effects of the precision of temporal synchrony as revealed by gamma-band oscillations.. Neuropsychologia.

[41] Giard M. H, Peronnet F (1999). Auditory-visual integration during multimodal object recognition in humans: a behavioral and electrophysiological study.. J Cogn Neurosci.

[42] Mishra J, Martinez A, Sejnowski T. J, Hillyard S. A (2007). Early cross-modal interactions in auditory and visual cortex underlie a sound-induced visual illusion.. J Neurosci.

[43] Driver J, Spence C (2000). Multisensory perception: beyond modularity and convergence.. Curr Biol.

[44] Schroeder C. E, Lakatos P, Kajikawa Y, Partan S, Puce A (2008). Neuronal oscillations and visual amplification of speech.. Trends Cogn Sci.

[45] Kayser C, Logothetis N. K, Panzeri S (2010). Visual enhancement of the information representation in auditory cortex.. Curr Biol.

[46] Luo H, Poeppel D (2007). Phase patterns of neuronal responses reliably discriminate speech in human auditory cortex.. Neuron.

[47] Simoncelli E. P, Olshausen B. A (2001). Natural image statistics and neural representation.. Annu Rev Neurosci.

[48] Karklin Y, Lewicki M. S (2009). Emergence of complex cell properties by learning to generalize in natural scenes.. Nature.

[49] Logothetis N. K, Pauls J, Augath M, Trinath T, Oeltermann A (2001). Neurophysiological investigation of the basis of the fMRI signal.. Nature.

[50] Kayser C, Montemurro M. A, Logothetis N. K, Panzeri S (2009). Spike-phase coding boosts and stabilizes information carried by spatial and temporal spike patterns.. Neuron.

[51] Montemurro M. A, Rasch M. J, Murayama Y, Logothetis N. K, Panzeri S (2008). Phase-of-firing coding of natural visual stimuli in primary visual cortex.. Curr Biol.

[52] Salinas E, Sejnowski T. J (2001). Correlated neuronal activity and the flow of neural information.. Nat Rev Neurosci.

[53] Makeig S, Westerfield M, Jung T. P, Enghoff S, Townsend J (2002). Dynamic brain sources of visual evoked responses.. Science.

[54] Fiser J, Chiu C, Weliky M (2004). Small modulation of ongoing cortical dynamics by sensory input during natural vision.. Nature.

[55] Schaefer A. T, Angelo K, Spors H, Margrie T. W (2006). Neuronal oscillations enhance stimulus discrimination by ensuring action potential precision.. PLoS Biol.

[56] Lakatos P, Karmos G, Mehta A. D, Ulbert I, Schroeder C. E (2008). Entrainment of neuronal oscillations as a mechanism of attentional selection.. Science.

[57] Lakatos P, O'Connell M. N, Barczak A, Mills A, Javitt D. C (2009). The leading sense: supramodal control of neurophysiological context by attention.. Neuron.

[58] Theunissen F. E, Sen K, Doupe A. J (2000). Spectral-temporal receptive fields of nonlinear auditory neurons obtained using natural sounds.. J Neurosci.

[59] Smith E. C, Lewicki M. S (2006). Efficient auditory coding.. Nature.

[60] Butts D. A, Weng C, Jin J, Yeh C. I, Lesica N. A (2007). Temporal precision in the neural code and the timescales of natural vision.. Nature.

[61] Shannon R. V, Zeng F. G, Kamath V, Wygonski J, Ekelid M (1995). Speech recognition with primarily temporal cues.. Science.

[62] Poeppel D (2003). The analysis of speech in different temporal integration windows: cerebral lateralization as ‘asymmetric sampling in time.’. Speech Communication.

[63] Hickok G, Poeppel D (2007). The cortical organization of speech processing.. Nat Rev Neurosci.

[64] Giraud A. L, Kleinschmidt A, Poeppel D, Lund T. E, Frackowiak R. S (2007). Endogenous cortical rhythms determine cerebral specialization for speech perception and production.. Neuron.

[65] Chandrasekaran C, Trubanova A, Stillittano S, Caplier A, Ghazanfar A. A (2009). The natural statistics of audiovisual speech.. PLoS Comput Biol.

[66] Canolty R. T, Edwards E, Dalal S. S, Soltani M, Nagarajan S. S (2006). High gamma power is phase-locked to theta oscillations in human neocortex.. Science.

[67] Ghazanfar A. A, Logothetis N. K (2003). Neuroperception: facial expressions linked to monkey calls.. Nature.

[68] Fairhall S. L, Macaluso E (2009). Spatial attention can modulate audiovisual integration at multiple cortical and subcortical sites.. Eur J Neurosci.

