# Supplementary figures and images for: Auditory Cortex Tracks Both Auditory and Visual Stimulus Dynamics Using Low-Frequency Neuronal Phase Modulation
Source: PLoS Biol. 2010 Aug 10;8(8):e1000445. doi: 10.1371/journal.pbio.1000445 (PMC2919416; doi:10.1371/journal.pbio.1000445)

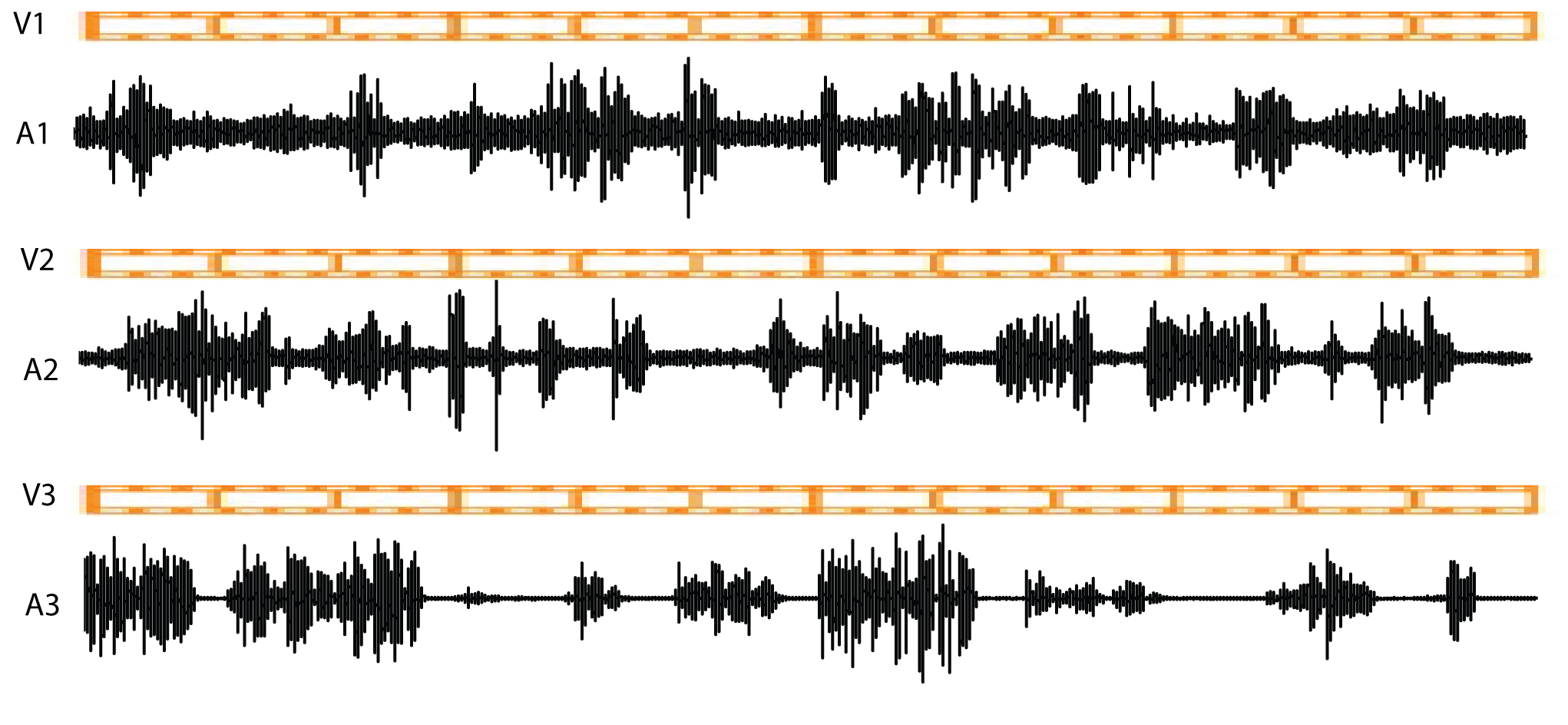

Supplement: Figure S1 — Audiovisual movie stimulus illustration. Three matched audiovisual movie clip illustration (V1+A1, V2+A2, V3+A3). The three mixed audiovisual movie stimuli are mixtures of V2+A1, V1+A3, and V3+A2. (0.67 MB DOC) [file pbio.1000445.s001.tif]

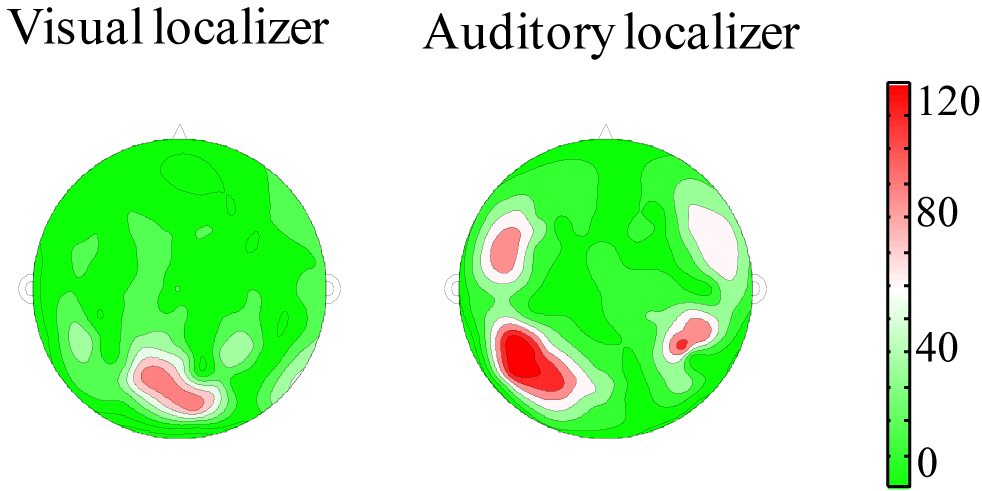

Supplement: Figure S2 — Channel localization (linked to Figures 2 , 3 , 4 , 5 , 6 , 7 in auditory and visual channels analysis). Auditory and visual localizer-based contour map for one representative subject. Red indicates a large absolute response value around the M100 peak latency (auditory localizer) and the M150 peak latency (visual localizer). Of the 157 recorded channels, 20 auditory and 20 visual channels were chosen based on the contour map for each subject—with no overlap allowed (i.e., the main analyses are based on spatially distinct sets of channels). Predictably, the visual localizer implicates occipital channels (both on the left and right of the midline), and the auditory localizer reflects the more anterior canonical (dipolar) distribution that has two channel groupings around a temporal lobe source (M100 dipole pattern). The color bar is in units of fT. (0.16 MB DOC) [file pbio.1000445.s002.tif]

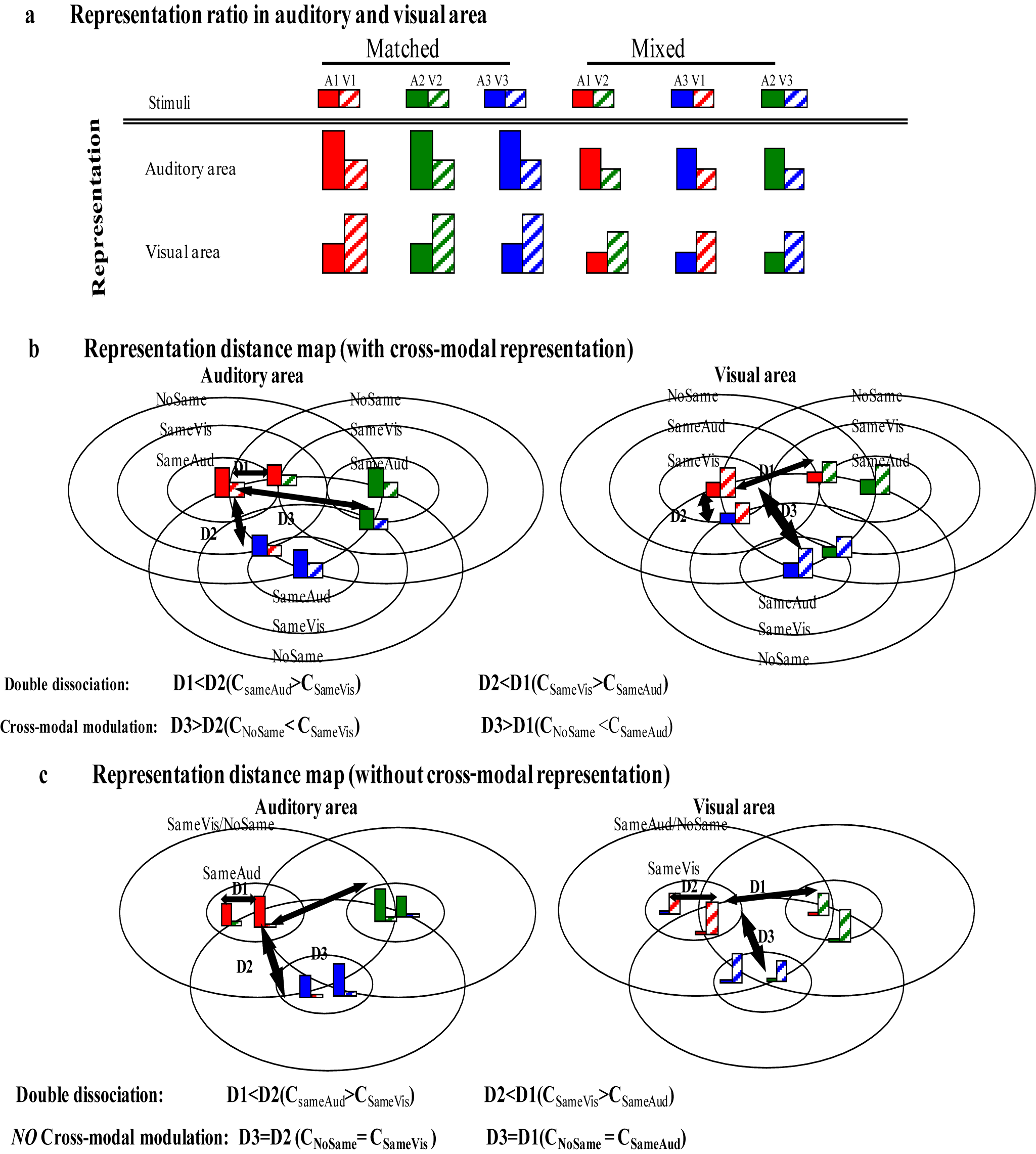

Supplement: Figure S3 — Cross-movie coherence analysis illustration (linked to Figure 3 and Figure 6 ). Illustration of the logic of cross-movie phase coherence analysis. In each of the six movie stimuli (first row of Figure S2), the solid bar represents the auditory stream and the hatched bar of the same color represents the corresponding visual stream. The middle and lower rows of Figure S2 indicate the hypothesized “representation ratio” of the stimulus in auditory and visual areas, respectively, in that the auditory stimulus dynamics will be more strongly represented in auditory cortex (solid bar) and the visual information (hatched bar) will be better represented in visual cortex. Crucially, if there exists direct modulation across sensory areas, the auditory area will also represent visual information, although to a lesser degree, and vice versa in the visual area. The figure illustrates an arbitrary hypothesized “representation distance” among the six movie stimuli in auditory and visual areas given the representation ratios in 2a. In this visualization, the distance between any two items corresponds to the similarity of the representation of the two movies, indicated by the arrow length between them (shorter distance means higher degree of similarity). D1, D2, and D3 correspond to the representation distance between one specific stimulus in the Matched group (A1V1 stimulus, for example) and the corresponding SameAud (A1V2), SameVis (A3V1), and NoSame (A2V3) counterparts in the Mixed group, respectively. A cross-modal representation results in the D2<D3 prediction for the auditory area and the D1<D3 prediction in the visual area. For example, the additional representation of visual information (hatched bar) in the auditory area makes the SameVis pair representation (D2) more similar (they both contain the representation for the same movie) compared to the NoSame pair. In contrast, as shown in Figure S2c, if there is no significant cross-modal representation (either no or an ineffect [file pbio.1000445.s003.tif]
