# Supplementary material for: Auditory Cortex Tracks Both Auditory and Visual Stimulus Dynamics Using Low-Frequency Neuronal Phase Modulation
Source: PLoS Biol. 2010 Aug 10;8(8):e1000445. doi: 10.1371/journal.pbio.1000445 (PMC2919416; doi:10.1371/journal.pbio.1000445)
Supplement: Text S1 — Cross-trial phase coherence versus traditional coherence analysis. Clarifying our cross-movie analysis, in comparison to traditional cross-channel coherence analysis. (0.03 MB DOC) [file pbio.1000445.s004.doc]

The cross-trial phase and power coherence measures employed in our analyses are motivated by but not identical to traditional coherence analysis, which is a measure of locking (in power or in phase) between two signals as a function of frequency:

$$C_{xy}(f)=\frac{S_{xy}(f)}{\sqrt{(S_{x}(f)S_{y}(f))}}$$

In traditional coherence analysis, $S_{x}(f)$ and $S_{y}(f)$represent signals from different areas (e.g., different channels in MEG/EEG studies, different neuron groups in neurophysiological studies), and the coherence values indicate how correlated the responses from two areas are (see review by Senkowski et al.). Our cross-trial coherence analysis is based on a different perspective. We are exploring the coherence pattern *within channels* instead of across channels; that is, we are interested in the extent to which an audiovisual stream can elicit a reliable and discriminable response pattern in a single channel. To address this question, we quantified the cross-trial coherence for each specific audiovisual stimulus (larger coherence indicates reliable response pattern) in each channel. In addition, we are interested in exploring how similar the response patterns elicited by two different movie stimuli are, by performing a cross-movie coherence analysis that is also single-channel-based. For example, for a specific movie, the movie sharing the same auditory input but different visual input (SameAud) should elicit more similar response in auditory channels than the SameVis movie stimulus. The cross-movie coherence analysis is performed by grouping all the 15 trials for movie1 and all the 15 trials for movie2, and then calculating the cross-30-trial coherence, which can reveal how similar the responses for the two movies are. The cross-movie coherence is thus quite similar to the cross-trial coherence, which is to quantify how similar (or how reliable) the responses in single trial for same movie stimulus are (see Figure 1ab for more details). In sum, the cross-modal modulation effects in our data are not based on conventional cross-channel coherence analyses that demonstrate response coherence between auditory and visual channels, but derive from the observation that visual input can robustly modulate the auditory channel response pattern and that auditory input can reliably influence activity in visual channels. Our approach is similar to the one taken in animal studies that show that an auditory neuron can be modulated by nonauditory input (Bizley et al., 2006, Kayser et al., 2005, 2008, Fu et al., 2003, Lakatos et al., 2007).
