# Supplementary material for: Auditory Cortex Tracks Both Auditory and Visual Stimulus Dynamics Using Low-Frequency Neuronal Phase Modulation
Source: PLoS Biol. 2010 Aug 10;8(8):e1000445. doi: 10.1371/journal.pbio.1000445 (PMC2919416; doi:10.1371/journal.pbio.1000445)
Supplement: Text S2 — Ruling out “leaking” induced cross-modal modulation. Control analysis. (0.03 MB DOC) [file pbio.1000445.s005.doc]

One argument that might challenge our view of genuine cross-modal phase modulation is that the auditory MEG channels significantly capture the magnetic fields generated by occipital neuronal sources, even if reduced in strength compared to the magnetic fields over temporal sources (and vice versa for the visual channels). To evaluate this potential alternative explanation, we performed three types of control analyses. **First**, we performed the entire analysis again, in only 10 auditory and 10 spatially non-overlapping visual channels (instead of 20) to render the ‘leaking’ possibility less likely from a purely MEG-geometric perspective (distant channels), and we still observed modality-specific tracking and cross-modal tracking in theta phase pattern, as we before. **Second**, we inspected the correlation coefficient between the phase tracking for auditory streams and that for visual streams in auditory and visual areas separately. We observed significant positive correlation between $C_{sameAud}$ and $C_{sameVis}$ in both of the auditory channels (Pearson’s correlation, r=0.92, p<0.001) and visual channels (r=0.79, p<0.001), indicating that the sensory channels displaying more robust same-modality stream tracking also show stronger tracking to the other modality streams. This control analysis result cannot be accounted for by the ‘leaking activity’ explanation and further supports the conclusion that our data are best explained by cross-modal modulation. **Third**, we examined the correlation coefficient between the responses to the visual localizer pretest ($A_{vis}$) and the cross-trial phase coherence for SameVis movie pairs ($C_{sameVis}$) in auditory channels. If the ability of these auditory channels to track visual stimuli (the cross-trial phase coherence for SameVis) is caused by a leaking response from visual cortex, the two parameters should show a stronger correlation ($CV_{vis,sameVis}$), since the most visually responsive channels in auditory cortex should be more driven by the visual cortical activities, in turn showing higher phase tracking to visual stimuli. The results for auditory channels showed no significant correlation in $CV_{vis,sameVis}$ (r=0.035, p=0.7), confirming that ‘leaking activity’ cannot well account for the observed tracking to visual streams in auditory areas, whereas $CV_{aud,sameAud}$ and $CV_{aud,sameVis}$ showed significant correlation (r=0.23, p=0.01; r=0.22 , p=0.014), suggesting again that it is the most representative auditory channels that display robust tracking to both auditory and visual streams, further ruling out the ‘leaking’ explanation. In sum, the control analyses suggest that the *observed cross-modal phase tracking is not due to cross-channel leaking*, and the most representative auditory/visual channels (instead of the putative ‘cross-modal leaking’ channels) contribute most to both auditory and visual stream tracking, and performed both ‘within-modal’ and ‘cross-modal’ phase tracking.
